# Cardiopulmonary Capacity in Children During Exercise Testing: The Differences Between Treadmill and Upright and Supine Cycle Ergometry

**DOI:** 10.3389/fphys.2019.01440

**Published:** 2019-11-29

**Authors:** Tonje Reitan Forbregd, Michelle Arthy Aloyseus, Ansgar Berg, Gottfried Greve

**Affiliations:** ^1^Department of Clinical Science, University of Bergen, Bergen, Norway; ^2^Department of Pediatrics and Adolescents Medicine, Haukeland University Hospital, Bergen, Norway; ^3^Department of Heart Diseases, Haukeland University Hospital, Bergen, Norway

**Keywords:** cardiopulmonary exercise testing, children’s physiology, spirometry, cardiac output, peak V̇O_2_, cardiopulmonary capacity, exercise testing

## Abstract

**Background/Hypothesis:** Cardiopulmonary exercise testing (CPET) is used in the assessment of function and prognosis of cardiopulmonary health in children with cardiac and pulmonary diseases. Techniques, such as cardiac MRi, and PET-scan, can be performed simultaneously with exercise testing. Thus, it is desirable to have a broader knowledge about children’s normal cardiopulmonary function in different body postures and exercise modalities. The aim of this study was to investigate the effect of different body positions on cardiopulmonary function in healthy subjects performing CPETs.

**Materials and Methods:** Thirty-one healthy children aged 9, 12, and 15 years did four CPETs: one treadmill test with a modified Bruce protocol and three different bicycle tests with different body postures, sitting, tilted 45°, and lying flat (0°). For the bicycle tests, a 20-watt ramp protocol with a pedal frequency of 60 ± 5 rotations per minute was used. Continous ECG and breath-by-breath ${\dot{\text{V}}\text{O}}_{2}$ measurements was done throughout the tests. Cardiac structure and function including aortic diameter were evaluated by transthoracic echocardiography prior to the tests. Doppler measurements of the blood velocity in the ascending aorta were measured prior to and during the test. Prior to every test, the participants performed pulmonary function tests with maximum voluntary ventilation test.

**Results:** There is a significantly (*p* < 0.05) lower peak ${\dot{\text{V}}\text{O}}_{2}$ in all bicycle tests compared with the treadmill test. There is lower corrected peak ${\dot{\text{V}}\text{O}}_{2}$ (ml kg^−0.67^ min^−1^), but not relative peak ${\dot{\text{V}}\text{O}}_{2}$ (ml kg^−1^ min^−1^), in the supine compared with the upright bicycle test. There are no differences in peak stroke volume or cardiac output between the bicycle modalities when calculated from aortic blood flow. Peak heart rate decreases from both treadmill to upright bicycle and from upright bicycle to the supine test (0°).

**Conclusion:** There are no differences in peak cardiac output between the upright bicycle test and supine bicycle tests. Heart rate and corrected peak ${\dot{\text{V}}\text{O}}_{2}$ are lower in the supine test (0°) than the upright bicycle test. In the treadmill test, it is a higher absolute and relative peak ${\dot{\text{V}}\text{O}}_{2}$. Despite the latter differences, we are convinced that both upright and supine bicycle tests are apt in the clinical setting when needed.

## Introduction

Cardiopulmonary exercise testing (CPET) is important in the assessment of function and prognosis of children’s cardiopulmonary health in a clinical setting (Albouaini et al., 2007; Leclerc, 2017, Stephens, 2017). CPET is important in diagnostics of diseases such as exercise-induced laryngeal obstruction (EILO) (Johansson et al., 2015) and exercise-induced asthma or bronchial obstruction (EIA/EIB) (Anderson and Kippelen, 2012). In addition, CPET is used for monitoring function in children with congenital and acquired heart diseases (Carano et al., 1999; Hebert et al., 2014) as well as in children and adolescents with cerebral palsy (Verschuren and Balemans, 2015). A treadmill or an upright bicycle test is the most used exercise modality for CPET (Cooper, 1995).

There is a growing interest in conduction of simultaneous MRI-scanning, PET-scanning, or echocardiography during a CPET (Gusso et al., 2012). By conducting these supplementary investigations, one may achieve improved overview of cardiopulmonary health during the CPET (Chesler and Stein, 2004; Cullen and Pellikka, 2011; Barber et al., 2016). This is possible if the test subject is fixed and in a supine body position during the tests. In addition, a supine bicycle test will accommodate a better way to perform CPETs for patients who are unable to perform the test in an upright body position due to physical disabilities. Thus, advantages and disadvantages of different body positions during CPETs have been investigated in later years.

In the literature, there is no generally accepted definition of exercise capacity. However, peak oxygen consumption (peak ${\dot{\text{V}}\text{O}}_{2}$) is commonly used as an indicator of physical fitness and exercise capacity (Shuleva et al., 1990; Armstrong et al., 1991; Figueroa-Colon et al., 2000; Fletcher et al., 2001; LeMura et al., 2001; American Thoracic Society, 2003; Johnston et al., 2005; Armstrong and Welsman, 2007).

Due to a relatively smaller cross section area of leg muscles (Bar-Or, 1983; Lexell et al., 1992), most children will have a lower peak ${\dot{\text{V}}\text{O}}_{2}$ on a bicycle than on a treadmill. Previous studies have found a significant lower peak ${\dot{\text{V}}\text{O}}_{2}$ when using the upright bicycle for CPET in young children (Armstrong et al., 1991; LeMura et al., 2001). These results correlate with similar research done in adults (Quinn et al., 1995; Egaña et al., 2006).

There is some knowledge of children and adolescent’s normal cardiopulmonary response to CPET in the supine bicycle positions (Moller et al., 2009), but there is still a need to further investigate the cardiopulmonary response in different body positions.

This study anticipates that both the gravitation and the changes of hemodynamic conditions will affect the cardiopulmonary performance of the children in a negative direction in CPETs in the supine body posture. This study aimed to assess cardiopulmonary responses in CPETs in four different body postures, performed by healthy children. In addition, the differences in cardiopulmonary capacity in CPETs between age groups, ranging from children to adolescents was studied. This was to study differences in cardiopulmonary capacity before and during puberty, as it is known that both blood pressure and muscle mass change during puberty.

## Method

### Subjects

Three cohorts of children, born in 1999, 2002, and 2005, were included in the study. They were tested in 2014 and hence, at ages of 9, 12, and 15 years, respectively. Ten children in the two oldest age groups and 11 children in the youngest age group were included. The children were recruited from schools in Bergen, Norway. The subjects were excluded if they had history of smoking, cardiovascular or lung disease, family history of cardio-pulmonary diseases, and physical difficulties performing the tests. The test subjects served as their own controls in the comparison of the different test positions. Lean body mass was calculated with the Peters equation (Peters et al., 2011) for the 9- and 12-year-olds and the Boer equation for the 15-year-olds (Table 1; Boer, 1984).

**Table 1:** Calculated anthropometric values for the test groups.

	Height ± SD (cm)	Weight ± SD (kg)	BMI ± SD (kg/m^2^)	LBM ± SD (kg)	Number of participants
9-year-olds	139.5 ± 3.9	32.7 ± 4.6	16.8 ± 2.5	27.7 ± 2.5	11
12-year-olds	158.0 ± 6.6*	47.6 ± 8.8*	18.9 ± 2.2	38.7 ± 1.8*	10
15-year-olds	169.6 ± 6.9*^,^^†^	55.7 ± 7.8^*^^,^^†^	19.3 ± 1.8*	47.4 ± 5.7*	10

*BMI, body mass index; LBM, lean body mass; SD, standard deviation*.

* *Significantly different from the 9-year-old calculated mean*.

† *Significantly different from the 12-year-old calculated mean*.

Prior to the start of the study, the participants’ parents signed informed written consents. The study was approved by the Regional Committee for Medical and Health Research in Western Norway (REK Vest 2014/1056).

### Exercise Test

The tests were performed in a randomized order and conducted at the Heart and Lung Test Laboratory at The Department of Child and Adolescents Medicine at Haukeland University Hospital, Bergen, Norway.

#### Exercise Protocol

**Figure 1:**
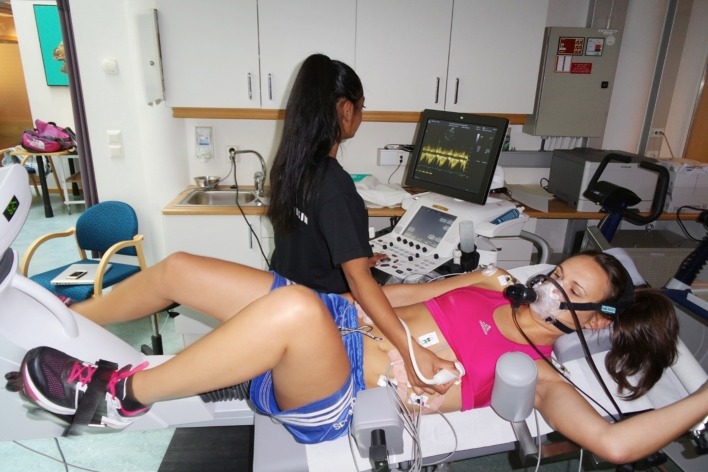
A 0° supine bicycle set-up illustrated, here with an adult volunteer. Informed, written consent for publication was obtained from the individuals in this photograph.

All participants performed the following tests: (1) treadmill CPET, (2) ergometer upright bicycle (sitting) CPET, (3) CPET in a supine bike tilted at 45° to the floor, and (4) CPET in a supine bike tilted at 0° to the floor (illustrated in Figure 1). The tests were conducted with at least 24 h between them. The test subjects were not instructed to refrain from their normal day activities or diet.

For the upright bicycle CPET, an electromagnetic resistance seeking ergometer bicycle (Corival, Lode B.V., Groningen, The Netherlands) was used. An electromagnetic resistance seeking tilt bicycle (Ergoselect 1,200, ergoline, Bitz, Germany) was used in the 0° and 45° bicycle tests. For all three bicycle tests, a 20-watt (W) ramp protocol (Buys et al., 2012; Armstrong and Welsman, 2019) was utilized. In the 20-W protocol, the resistance starts with a resistance of 20 W and increases with 2 W every fifth second, i.e., 20 W every minute. It was of importance to keep the length of the test within a timeframe so that the children did not get impatient. The participants were instructed to keep a speed of 60 rotations per minute (rpm) with a range between 55 and 65 rpm (Blanchard et al., 2018), which in our experience is the range that suits most children well.

The incremental peak treadmill (ELG 70, Woodway, Weil am Rhein, Germany) exercise test was executed with a modified and computerized Bruce protocol identical for all subjects (Duff et al., 2017). Speed and elevation were gradually increased every 60 s (see Supplementary Table S1), starting from an initial slow-walking phase (Cumming et al., 1978; Paridon et al., 2006; Clemm et al., 2012).

The exercise test was considered to have reached peak level when the participant indicated subjective exhaustion, preferably supported by a plateau in ${\dot{\text{V}}\text{O}}_{2}$ or heart rate (HR) response (Paridon et al., 2006), or a respiratory exchange ratio (RER) higher than 1.1 (Rowland et al., 2017). Thus, the tests were not considered to be max tests, but rather peak tests. Direct breath-by-breath measurements were continuously monitored on the computer screen throughout the tests, to supervise the physiological response to exercise.

#### Cardiopulmonary Exercise Testing Measurements

Prior to the start of each test, the participants did a forced spirometry test (Vmax 29, SensorMedics, Yorba Linda, CA, USA) and a maximum voluntary ventilation (MVV) test. Variables of gas exchange and airflow were measured breath-by-breath with a facemask (Hans Rudolph Inc., Kansas City, MO, USA) connected to Oxycon pro®JLAB 5.x. version 1.0 (Jaeger®, Care Fusion, San Diego, CA, USA) set up with standard layout Vmax29 cardiopulmonary exercise unit CPET computer program (SensorMedics, Yorba Linda, CA, USA). The participants wore a mask with a digital TripleV-Volume sensor. Cardiopulmonary measurements were averaged per 10 s. The highest value determined during the last 60 s was used as peak value. Peak ${\dot{\text{V}}\text{O}}_{2}$ was reported as ml min^−1^, ml kg^−1^ min^−1^, or as a corrected value, which has been used by some groups (ml kg^−0.67^ min^−1^) (Zapletal, 1987; Pettersen and Fredriksen, 2003; Wasserman et al., 2005).

During the CPET, a 12-lead ECG (GE CardioSoft V6.51, General electric company, Fairfield, CT, USA) recorded heart activity simultaneously, and blood pressure was measured every 2 min with SunTech Tango+ (SunTech Medical, Morrisville, NC, USA).

#### Echocardiography

Echocardiography was performed using an ultrasound system with a 2.5-MHz transducer (Vivid E9, GE Vingmed, Canada). These measurements were performed by the same operator at every test. Prior to exercise testing, normal cardiac structure and function were confirmed. The internal aortic diameter was measured in the parasternal long- and short-axis at the valvular level. The aortic diameter was assumed to be constant throughout a cycle and during exercise as there is only a small increase in aortic root size at the aortic valve annulus (Iskandar and Thompson, 2013).

Thereafter, Doppler measurements were obtained from the ascending aorta at every minute during the bicycle tests, including the post-exercise period (5 min). In the supine tests, this was done by continuous-wave (CW) Doppler measurements at the aortic valve visualized in the four-chamber view. In the upright bicycle test, the velocity of ascending blood was measured by a two-dimensional continuous-wave transducer positioned in the suprasternal notch pointing toward the origin of the aortic root. The measurement from the suprasternal notch has proved to give an accurate measurement of aortic blood flow (Lima et al., 1983). Stroke volume (SV) was assessed by standard Doppler echocardiographic methods and estimated as the product of the mean velocity-time integral (VTI). VTI was calculated tracing the velocity curve contour across the aortic valve, and the end-point of each contour was marked by aortic valve closure. The best-defined spectral curves out of three were averaged every minute (Quiñones et al., 2002; Vignati and Cattadori, 2017). Cardiac output (CO) was calculated by multiplying SV with heart rate (HR) (Leyk et al., 1994).

### Statistics

SPSS 25 (IBM Corporation, Armonk, NY, USA) was used for all statistical analyses. Groups were checked for normality. Group means, standard deviation, and ranges were calculated as appropriate. *p* < 0.05 was considered significant.

A repeated-measures one-way ANOVA with Greenhouse-Geisser correction was used to investigate the mean differences in ventilatory and respiratory variables. *Post hoc* tests using the Bonferroni correction was used to further explore the differences in mean in the different body positions for main outcome variables.

For investigation of mean differences between age groups, a one-way ANOVA for independent measures was performed for each ventilatory and respiratory variable.

Using a paired sample *t*-test, the difference between rest values and peak values for the variables SV, HR, and CO for each test person in each age group was investigated.

### New Equipment

New and updated equipment, as well as software, had to be used for five subjects in the 9-year-olds group. This was due to renovation of the Children’s Hospital during the test period. Replacements of equipment included the treadmill (bari-mill, Woodway, Weil am Rhein, Germany), Jaeger® Vyntus CPX Canopy metabolic cart and SentrySuite® respiratory software platform (Jaeger®, CareFusion, San Diego, CA, USA), custo cardio 100 12-lead ECG recorder (Custo Med, GmbH, Ottobrunn, Germany), and Tango M2 blood pressure system (SunTech Medical, Morrisville, NC, USA). According to international recommendations, our exercise facilities are set up with biological controls. In biological controls, no systematical or significant alterations in variables between old and new equipment have been detected. Similarly, there are no differences (Student’s *t*-test) in values between age-matched children tests on the new equipment compared with tests on the old equipment.

## Results

Main anthropometric variables are shown in Table 1. It is not segregated based on gender in the groups as the participants function as their own control. Analysis shows significant mean differences in height between all age groups (139.5 ± 3.9, 158.0 ± 6.6, 169.6 ± 6.9 cm). The weight does not differ significantly between the 9- and 12-year-olds, but it does between the other groups (32.7 ± 4.6, 47.6 ± 8.8, 55.7 ± 7.8 kg). Also, only the mean difference in body mass index (BMI) between 9- and 15-year-olds is significant (16.8 ± 2.5, 19.3 ± 1.8).

### Pulmonary Function Test

Forced vital capacity (FVC) and forced expiratory volume in 1 s (FEV1) are higher in standing and sitting positions compared with the lying position (0° supine) (FVC; 3.2 ± 1.2 vs. 3.0 ± 1.2 L), (FEV1; 2.6 ± 0.9 vs. 2.4 ± 0.9 L), and lower maximal voluntary ventilation (MVV) in 45° supine position than in the sitting and 45° tilted position (85 ± 31 vs. 92 ± 35 L). All values are presented in Table 2.

**Table 2:** Calculated means for spirometry variables obtained prior to the exercise tests.

	FEV1 (L) mean ± SD	FVC (L) mean ± SD	MVV (L/min) mean ± SD
Treadmill/standing	2.6 ± 0.9	3.2 ± 1.2	92 ± 35
UB/sitting	2.7 ± 0.9	3.4 ± 1.3	92 ± 33
45° SB	2.4 ± 0.8*^,^^†^	3.1 ± 1.2^†^	85 ± 31*^,^^†^
0° SB	2.4 ± 0.9*^,^^†^	3.0 ± 1.2*^,^^†^	81 ± 37^*^^,^^†^

UB, upright bicycle; SB, supine bicycle; FVC, forced vital capacity; FEV1, forced expiratory volume in 1 s; MVV, maximum voluntary ventilation. *N* = 30.

* *Significantly different to treadmill test*.

† *Significantly different to upright bicycle test*.

### Breath-by-Breath Measurements

Peak ${\dot{\text{V}}\text{O}}_{2}$, reported in ml min^−1^ (absolute), ml kg^−0.67^ min^−1^ (corrected), and ml kg^−1^ min^−1^ (relative), are significantly higher during the treadmill exercise test compared with all three bicycle modalities (52.9 ± 7.6 vs. 43.0 ± 6.2 ml kg^−1^ min^−1^). It is not a difference between the bicycle modalities for relative peak ${\dot{\text{V}}\text{O}}_{2}$, but for absolute and corrected peak ${\dot{\text{V}}\text{O}}_{2}$ there is a decrease from upright bicycle to the 0° supine test (Table 3). When accounted for lean body mass (LBM) it is a difference between the upright bicycle and the supine bicycle (53.7 ± 7.9 vs. 51.0 ± 6.6) as well. There is a significantly higher mean minute ventilation (VE) and tidal volume (VT) during the treadmill test than in the other test modalities (85.5 ± 27.7 vs. 69.8 ± 26.4) (Table 4).

**Table 3:** Calculated means for peak oxygen uptake in all test groups.

	Peak $\dot{V}O_{2}$ (ml min^−1^) mean ± SD	Peak $\dot{V}O_{2}$ (ml kg^−1^ min^−1^) mean ± SD	Peak $\dot{V}O_{2}$ (ml kg^−0.67^ min^−1^) mean ± SD	Peak $\dot{V}O_{2}$ adjusted for LBM (ml kg^−1^ min^−1^) mean ± SD
Treadmill	2,373 ± 710	52.9 ± 7.6	184.0 ± 30.5	62.6 ± 7.8
Upright bicycle	2050 ± 683*	45.3 ± 7.3*	158.1 ± 31.2*	53.7 ± 7.9*
45° supine	2014 ± 714*	44.1 ± 7.6*	154.5 ± 33.7*	52.4 ± 8.5*
0° supine	1945 ± 622*^,^^†^	43.0 ± 6.2*	150.2 ± 27.3*^,^^†^	51.0 ± 6.6^*^^,^^†^

*peak* ${\dot{V}O}_{2}$, *peak oxygen consumption; LBM, lean body mass. N = 31*.

* *Significantly different to treadmill test*.

† *Significantly different to upright bicycle test*.

**Table 4:** Calculated means for ventilatory variables in all test groups.

	HR_peak_ (beats/min) ± SD	VE (L/min) mean ± SD	VT (L) mean ± SD	RER (CO_2_ production/O_2_ uptake) mean ± SD
Treadmill	199 ± 8	85.5 ± 27.7	1.5 ± 0.1	1.18 ± 0.1
Upright bicycle	182 ± 10*	77.2 ± 30.9*	1.4 ± 0.1*	1.14 ± 0.1
45° supine	176 ± 15*	71.7 ± 28.0*	1.3 ± 0.1*^,^^†^	1.13 ± 0.1
0° supine	173 ± 16*^,^^†^	69.8 ± 26.4*	1.3 ± 0.1*^,^^†^	1.12 ± 0.1^*^

*HR, heart rate; VE, ventilation; VT, tidal volume. N = 31*.

* *Significantly different to treadmill test*.

† *Significantly different to upright bicycle test*.

**Figure 2:**
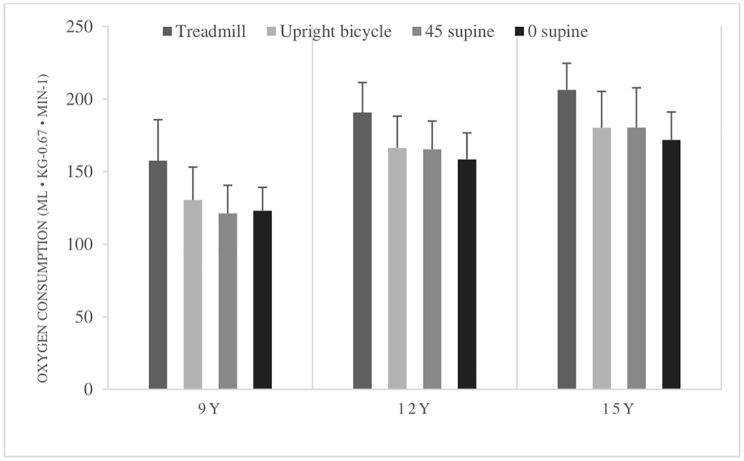
Peak oxygen consumption (peak ${\dot{V}O}_{2}$) (ml kg^−1^ min^−1^) means with standard deviations in all body postures in all age groups. 9Y = 9-year-olds, 12Y = 12-year-olds, 15Y = 15-year-olds. There is a significant higher peak ${\dot{V}O}_{2}$ in the treadmill test compared with the bicycle tests, and higher in the upright bicycle test compared to the 0° supine test. Peak ${\dot{V}O}_{2}$ was higher in the 12- and 15-year-olds in all tests compared to the 9-year-olds. No differences in peak ${\dot{V}O}_{2}$ between 12-year-olds and 15-year-olds.

Both when comparing the peak ${\dot{\text{V}}\text{O}}_{2}$ given in ml kg^−1^ min^−1^ and peak ${\dot{\text{V}}\text{O}}_{2}$ given in ml kg^−0.67^ min^−1^, between the age groups, there is a significant increase between the 9-year-olds and 12-year-olds in all four different test positions. However, there is no significant increase in corrected peak ${\dot{\text{V}}\text{O}}_{2}$ from 12- to 15-year-olds. The different peak ${\dot{\text{V}}\text{O}}_{2}$ means for all tests and age groups are shown in Figure 2. Results of breath-by-breath measurements are presented in Tables 3, 4.

### Cardiac Measurements

HR_peak_ is higher in the treadmill tests compared with the bicycle tests (199 ± 8 vs. 173 ± 16 beats/min) (Table 4). Comparison of the three bicycle tests gives no differences in HR_peak_. There are small differences between the age group means for HR_peak_. Comparison of age groups shows significant changes in CO between 9- and 12-year-olds, as well as 9- and 15-year-olds in the three bicycle tests. No difference in HR between age groups.

There are no significant differences in SV at CO_peak_, or in CO_peak_ when comparing the different modalities (Table 5). There is a significantly higher mean HR at CO_peak_ in the upright bicycle test than in the 0° supine bicycle test (180 ± 10 vs. 171 ± 17 beats/min).

**Table 5:** Group means of cardiac output (CO), heart rate (HR), and stroke volume (SV) with standard deviations.

	SV at CO_peak_ (ml/beat) ± SD	HR at CO_peak_ (beats/min) ± SD	CO_peak_ (L/min) ± SD
Upright bicycle	95.5 ± 30	180 ± 10	17.1 ± 5.6
45° supine	98.0 ± 36	174 ± 15	17.2 ± 6.8
0° supine	97.9 ± 30	171 ± 17^*^	16.8 ± 5.6

* *Significantly different from upright bicycle value*.

Investigation of change in HR, CO, and SV from rest to CO_peak_ shows significant increase of all values, except for SV from rest to peak in the upright bicycle for the youngest group (Supplementary Figure S6).

## Discussion

In this study, there is a lower absolute, corrected, and relative peak ${\dot{\text{V}}\text{O}}_{2}$ in all bicycle tests compared with the treadmill test as well as lower absolute and corrected peak ${\dot{\text{V}}\text{O}}_{2}$ in the supine compared with the upright bicycle test. There are no differences in peak stroke volume or cardiac output between the bicycle modalities. Peak heart rate decreased from both treadmill to upright bicycle and from upright bicycle to the supine tests.

Other studies have previously reported that the differences in children’s peak ${\dot{\text{V}}\text{O}}_{2}$ with altered body position to be smaller when adjusted for lean body mass (LBM) (Vinet et al., 2003; Eiberg et al., 2005). On the contrary, this study shows an additional change in peak ${\dot{\text{V}}\text{O}}_{2}$ when adjusted for LBM, where the results from the 0° supine bicycle is significantly lower than the upright bicycle test. The same result is present in the absolute and corrected peak ${\dot{\text{V}}\text{O}}_{2}$ results, but not the relative peak ${\dot{\text{V}}\text{O}}_{2}$. Correction with exponential factor of 0.67 has been claimed to express the peak ${\dot{\text{V}}\text{O}}_{2}$ of adolescents more correctly. This has been discussed to be due to that the increase in peak ${\dot{\text{V}}\text{O}}_{2}$ in children and adolescents is masked by the increase in body mass with age (Pettersen and Fredriksen, 2003). With the exponential correction factor, there is a significant difference in mean peak ${\dot{\text{V}}\text{O}}_{2}$ when comparing the upright bicycle and 0° supine bicycle, which was not present when only expressing the peak ${\dot{\text{V}}\text{O}}_{2}$ relative to body weight. This may strengthen the argument that a correction factor is needed in evaluation of peak ${\dot{\text{V}}\text{O}}_{2}$ in children and adolescents.

### Pulmonary Function Tests

As previously reported in adults, this study also found lower spirometry values in the supine position than in the sitting and standing positions (Vilke et al., 2000; Naitoh et al., 2014). However, there is no decrease in FEV1 and FVC from the standing to the sitting posture (Pierson et al., 1976). A systematic review of pulmonary function tests with different body positions is showing somewhat conflicting results in the literature regarding the effect of different body positions (Katz et al., 2018). But in general, FEV1, FVC, FRC, maximal expiratory pressure (PEmax), maximal inspiratory pressure (PImax), and peak expiratory flow (PEF) values were higher in more erect positions. For subjects with tetraplegic SCI, FVC and FEV1 were higher in supine vs. sitting position. In our study, MVV is lower in the supine than in the sitting position in accordance with results shown in adults by Vilke et al. (2000).

### Ventilation During Exercise

Studies of ventilatory function during exercise have shown conflicting results. LeMura et al. reported a trend but no significant differences in VE in their 5- and 6-year-olds when comparing treadmill and upright bicycle (LeMura et al., 2001). Boileau et al. studied a group of 11- to 14-year-old boys, and did not find any differences in VE when achieved on a treadmill or an ergometer bicycle (Boileau et al., 1977).

### Age-Group Differences in Cardiopulmonary Capacity

When comparing the test modalities, there were significant differences for most of the ventilatory and respiratory variables. There is also a higher CO in the 12- and 15-year-olds compared with the 9-year-olds. This appears to be due to higher SV in the oldest groups, as the HR was without any significant changes throughout the age groups. Stroke volume is related to body surface area, which explains our results well.

An interesting aspect of the between-group analysis is that the differences in peak ${\dot{\text{V}}\text{O}}_{2}$, when corrected for weight, is less than the absolute value and when using the correction factor of 0.67. However, when correcting with a power of −0.67 instead of −1 as used by some groups, there is still no increase in peak ${\dot{\text{V}}\text{O}}_{2}$ from 12- to 15-year-olds. A possible explanation is that the relative increase in weight compared with the increase in peak ${\dot{\text{V}}\text{O}}_{2}$ is greater, and thus gives a smaller increase in weight-adjusted peak ${\dot{\text{V}}\text{O}}_{2}$. This has been suggested in earlier studies as well (Krahenbuhl et al., 1985; Pettersen and Fredriksen, 2003).

As there is a lack of increase in relative and corrected peak ${\dot{\text{V}}\text{O}}_{2}$, as well as CO, between 12- and 15-year-olds, our results indicate that most changes in peak ${\dot{\text{V}}\text{O}}_{2}$ and CO occur between 9 and 12 years of age.

### Cardiopulmonary Measurements

In adults, linearity between CO and ${\dot{\text{V}}\text{O}}_{2}$ has been shown throughout exercise tests (Thadani and Parker, 1978; De Cort et al., 1991). Similar linearity is visualized in Supplementary Figures S1–S3. It was previously suggested that the linearity might be affected by fitness level (Beck et al., 2006; Trinity et al., 2012), and a study has shown that this linearity ceases at a certain point in the exercise, where CO starts decreasing as peak ${\dot{\text{V}}\text{O}}_{2}$ is approached (Stringer et al., 2005). Figures in the supplementary show (Supplementary Figures S1–S3) a similar tendency of a flat, or decreasing, CO toward the end is observed. This is however not present in all tests.

**Figure 3:**
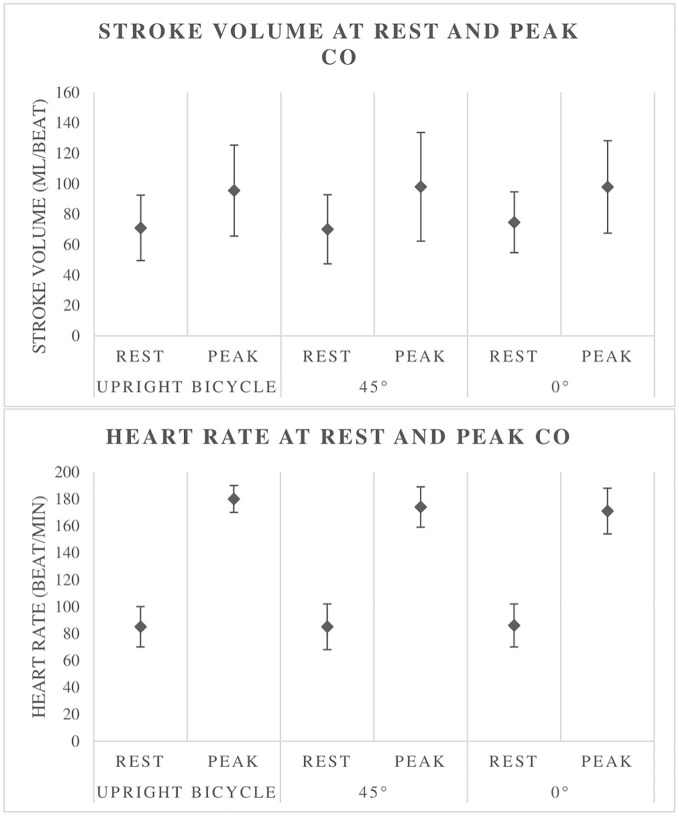
Stroke volume and heart rate at rest and at peak cardiac output (CO). Illustrates the increase in CO with exercise as well as little difference between the different tests.

There is an absence of differences in relative peak ${\dot{\text{V}}\text{O}}_{2}$ between bicycle CPETs in three different body positions. This is supported by the findings on CO, which is unchanged between the three different bicycle tests. However, there are some results suggesting a change in hemodynamics in the supine test. HR at CO_peak_, as well as absolute peak ${\dot{\text{V}}\text{O}}_{2}$_,_ is significantly lower in the supine tests than in the upright bicycle test (Tables 3–5). SVpeak also shows a somewhat higher mean value in the supine position, but this is not significant (Table 5, Figure 3). These findings may be explained by a higher preload in the supine position, a body posture which will give less work toward gravitational forces, thus improving venous backflow. Thus, the same CO was obtained at a lower HR.

Studies have investigated the hemodynamics with change of body position. Higginbotham and coworkers have reported individual differences with increase of end-diastolic volume in a supine position, which partly can explain the variation of SV changes (Higginbotham et al., 1986). It is also subjected that the decrease in intrathoracic pressure in an upright position might cause a shift of blood to the legs, and thus a reduced heart volume. Other hemodynamic considerations include a higher ventricular filling in a supine position, and a rise in HR and vascular resistance in an upright posture (Bevegard et al., 1960; Ray and Cureton, 1991).

Thadani et al. discussed that normal adult subjects have individual variation in stroke volume response to exercise in supine body positions, as studies have found conflicting results on this (Bevegard et al., 1960; Ross et al., 1965; Thadani and Parker, 1978). Also, CO has shown different results in studies, which underlines the variation in the normal population (Bevegard et al., 1960). Nevertheless, due to a lack of agreement of changes in SV and CO with altered body position, one can argue that the hemodynamics during exercise is not completely understood.

### Strength and Weaknesses

This study has mapped a small number of individuals and their performance in four different CPET modalities. Every participant conducted all four tests, which gave a strong basis for intra-individual comparisons. No segregation was made based on gender in the test groups. This might mask differences is peak values as sex differences have been reported in other studies. However, the main purpose of the test subjects was to serve as their own control when changing body posture and not to define absolute reference values. Conducting echocardiographic measurements, including VTI measurements, are challenging because of movement artifacts, especially at the end of the tests. The test personnel were the same throughout the project, providing less room for methodical human errors.

## Conclusion

This study does not find any differences in relative peak ${\dot{\text{V}}\text{O}}_{2}$ or peak cardiac output between the upright bicycle test and the supine bicycle tests. When correcting relative peak ${\dot{\text{V}}\text{O}}_{2}$ with an exponential factor of 0.67, we find a lower peak ${\dot{\text{V}}\text{O}}_{2}$ in the supine test (0°) compared to the upright bicycle test. Heart rate and absolute peak ${\dot{\text{V}}\text{O}}_{2}$ are lower in a supine than the upright bicycle test. In the treadmill test, higher absolute, corrected, and relative peak ${\dot{\text{V}}\text{O}}_{2}$ values are found compared to all bicycle tests. Our study supports the view that both upright and supine bicycle tests are apt in the clinical setting when needed.

## Data Availability Statement

Datasets are available on request. The anonymous raw data supporting the conclusions of this manuscript will be made available by the authors, depending on individual approval from the Data Protection Officer at Haukeland University Hospital.

## Ethics Statement

The studies involving human participants were reviewed and approved by Regional Etisk Komité (REK Vest 2014/1056). Written informed consent to participate in this study was provided by the participants’ legal guardian/next of kin. Written informed consent was obtained from the participants for the publication of any identifiable information or images.

## Author Contributions

AB and GG conceived of the presented idea, developed the theory, and supervised the findings of this work. TF and MA carried out the experiment. TF performed the analysis and interpretation and wrote the manuscript with support from MA, AB, and GG.

### Conflict of Interest

The authors declare that the research was conducted in the absence of any commercial or financial relationships that could be construed as a potential conflict of interest.
