# Supplementary material for: Cardiopulmonary Capacity in Children During Exercise Testing: The Differences Between Treadmill and Upright and Supine Cycle Ergometry
Source: Front Physiol. 2019 Nov 29;10:1440. doi: 10.3389/fphys.2019.01440 (PMC6897055; doi:10.3389/fphys.2019.01440)
Supplement: Supplementary file 1 [file Table_1.docx]

|  | Treadmill | | Upright bicycle | 45° supine | 0° supine |
| --- | --- | --- | --- | --- | --- |
|  | Speed (km/h) | Elevation (%) | Load (W) | Load (W) | Load (W) |
| Min 1 | 1.5 | 0 | 40 | 40 | 40 |
| Min 2 | 1.5 | 5 | 60 | 60 | 60 |
| Min 3 | 2.1 | 9 | 80 | 80 | 80 |
| Min 4 | 2.7 | 10 | 100 | 100 | 100 |
| Min 5 | 3.3 | 11 | 120 | 120 | 120 |
| Min 6 | 4.0 | 12 | 140 | 140 | 140 |
| Min 7 | 4.7 | 13 | 160 | 160 | 160 |
| Min 8 | 5.5 | 14 | 180 | 180 | 180 |
| Min 9 | 6.1 | 15 | 200 | 200 | 200 |
| Min 10 | 6.8 | 16 | 220 | 220 | 220 |
| Min 11 | 7.5 | 17 | 240 | 240 | 240 |
| Min 12 | 8.1 | 18 | 260 | 260 | 260 |

**Table S1:** Protocol for four tests, showing the increase for each minute.

# Supplementary Figures and Tables

**Figure 1**: Heart rate (HR), respiratory rate (RR), ventilation (VE), oxygen consumption (VO_2_) and cardiac output (CO) plotted against time elapsed for each of the four CPETs for 9-year-olds.

**Figure 2:** Heart rate (HR), respiratory rate (RR), ventilation (VE), oxygen consumption (VO_2_) and cardiac output (CO) plotted against time elapsed for each of the four CPETs for 12-year-olds.

**Figure 3**: Heart rate (HR), respiratory rate (RR), ventilation (VE), oxygen consumption (VO_2_) and cardiac output (CO) plotted against time elapsed for each of the four CPETs for 15-year-olds.

**Figure 4:** Continuous oxygen consumptions plotted towards heart rate (HR). Presented for each age group and all four tests.

**Figure 5:** Continuous oxygen consumption and ventilation throughout each test for all age groups.

**Figure 6:** Oxygen consumption plotted towards cardiac output for values throughout the tests. Shows linearity.
