# Supplementary figures and images for: Cardiopulmonary Capacity in Children During Exercise Testing: The Differences Between Treadmill and Upright and Supine Cycle Ergometry
Source: Front Physiol. 2019 Nov 29;10:1440. doi: 10.3389/fphys.2019.01440 (PMC6897055; doi:10.3389/fphys.2019.01440)

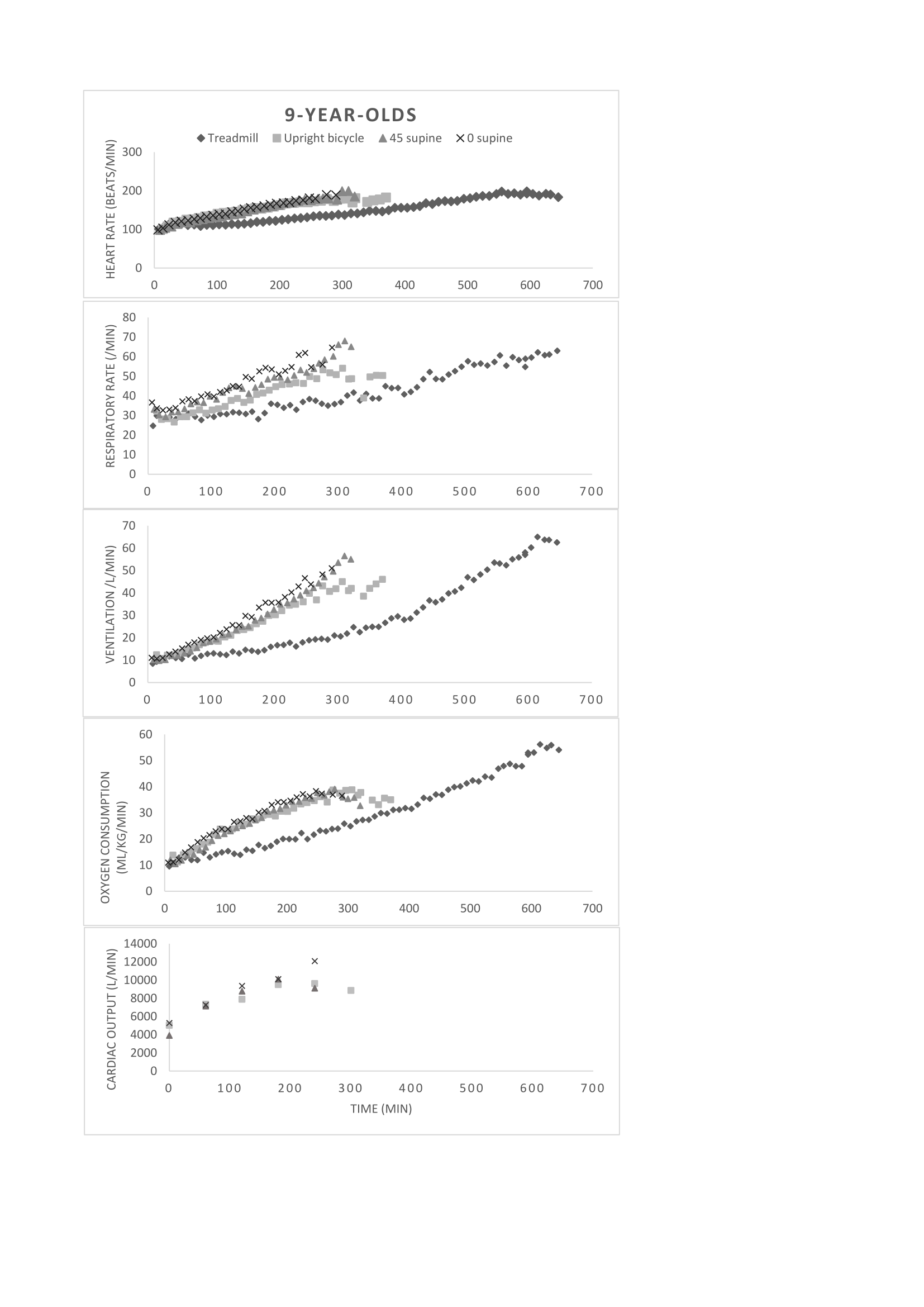

Supplement: Supplementary file 2 [file Image_1.tiff]

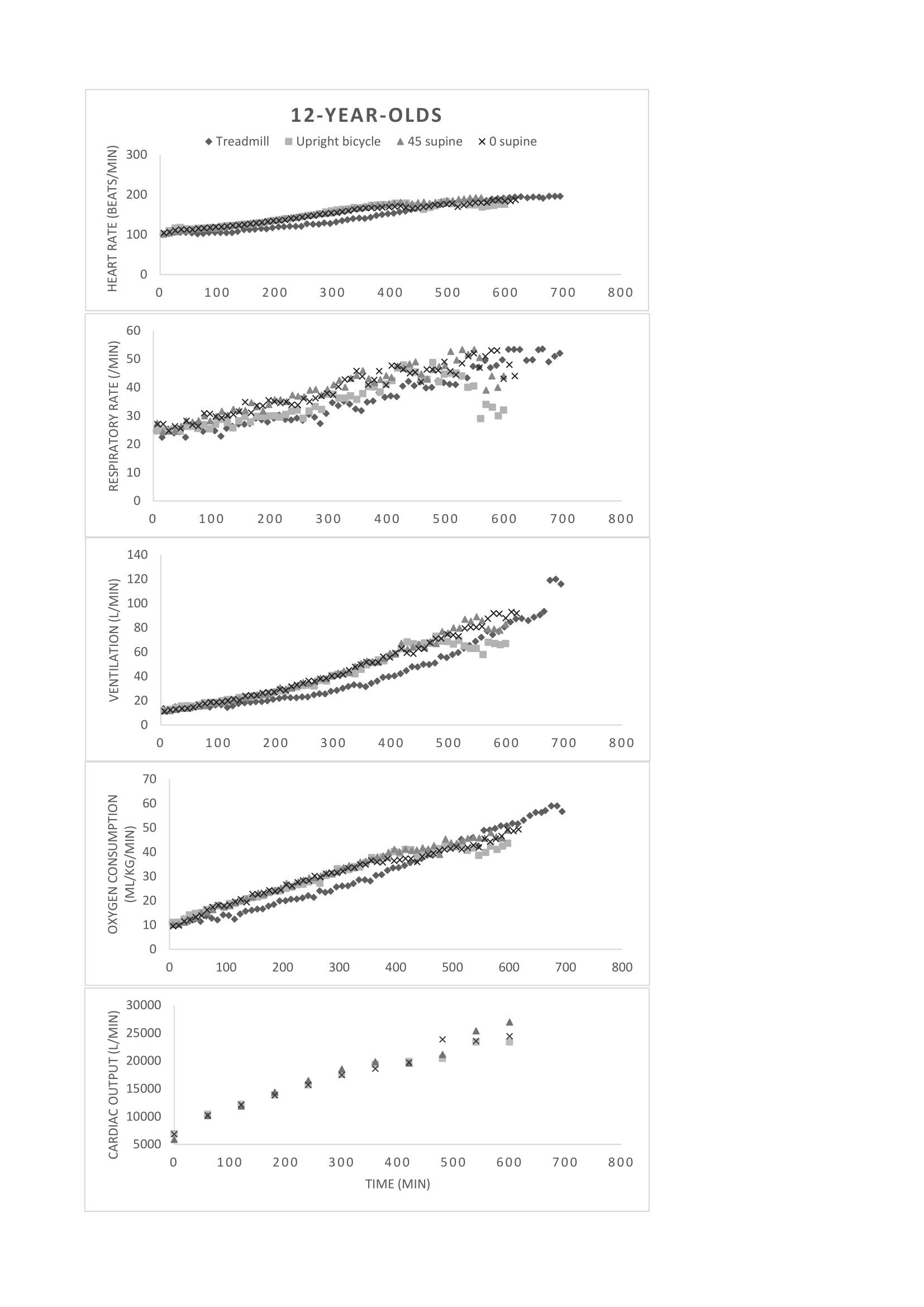

Supplement: Supplementary file 3 [file Image_2.tiff]

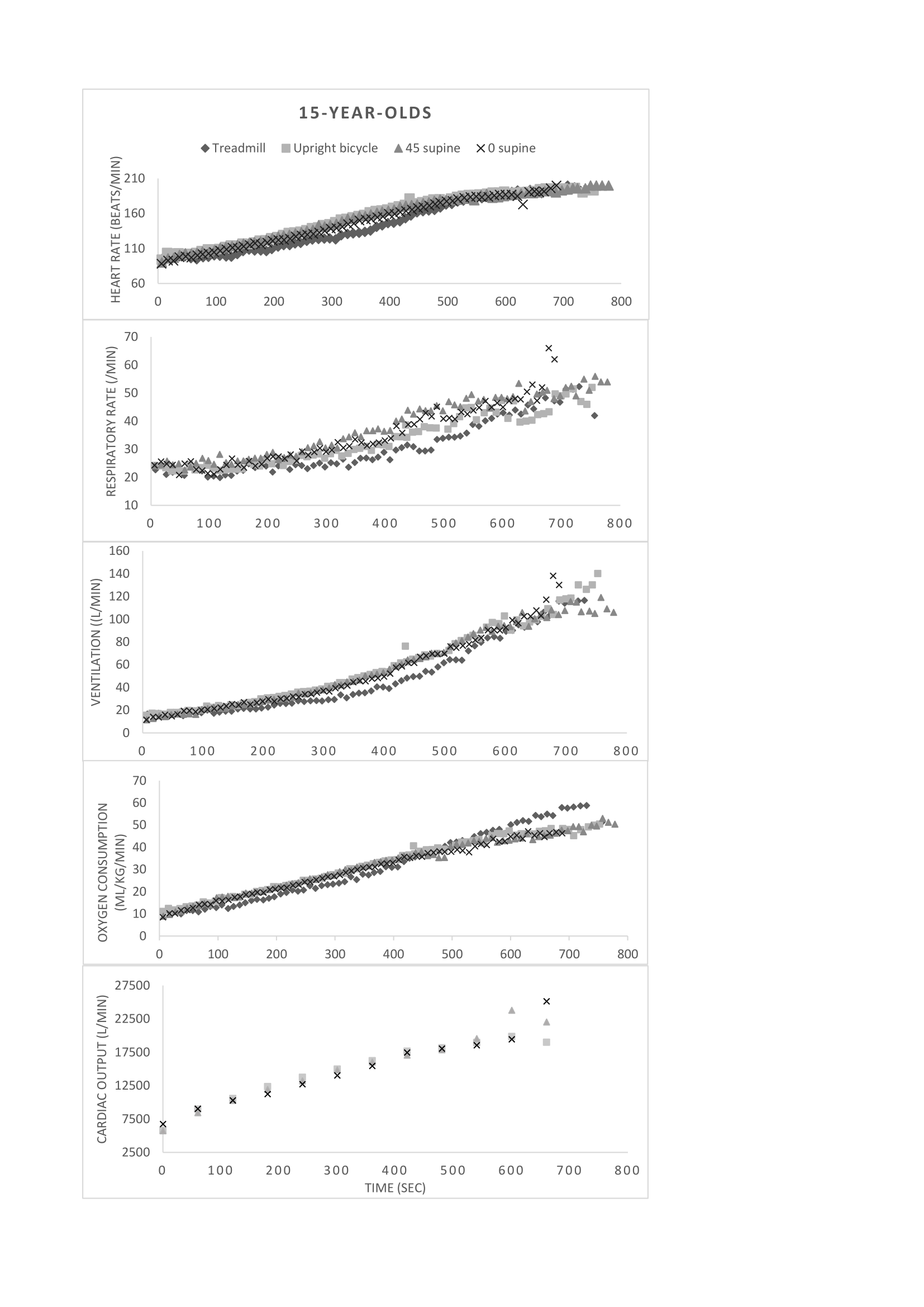

Supplement: Supplementary file 4 [file Image_3.tiff]

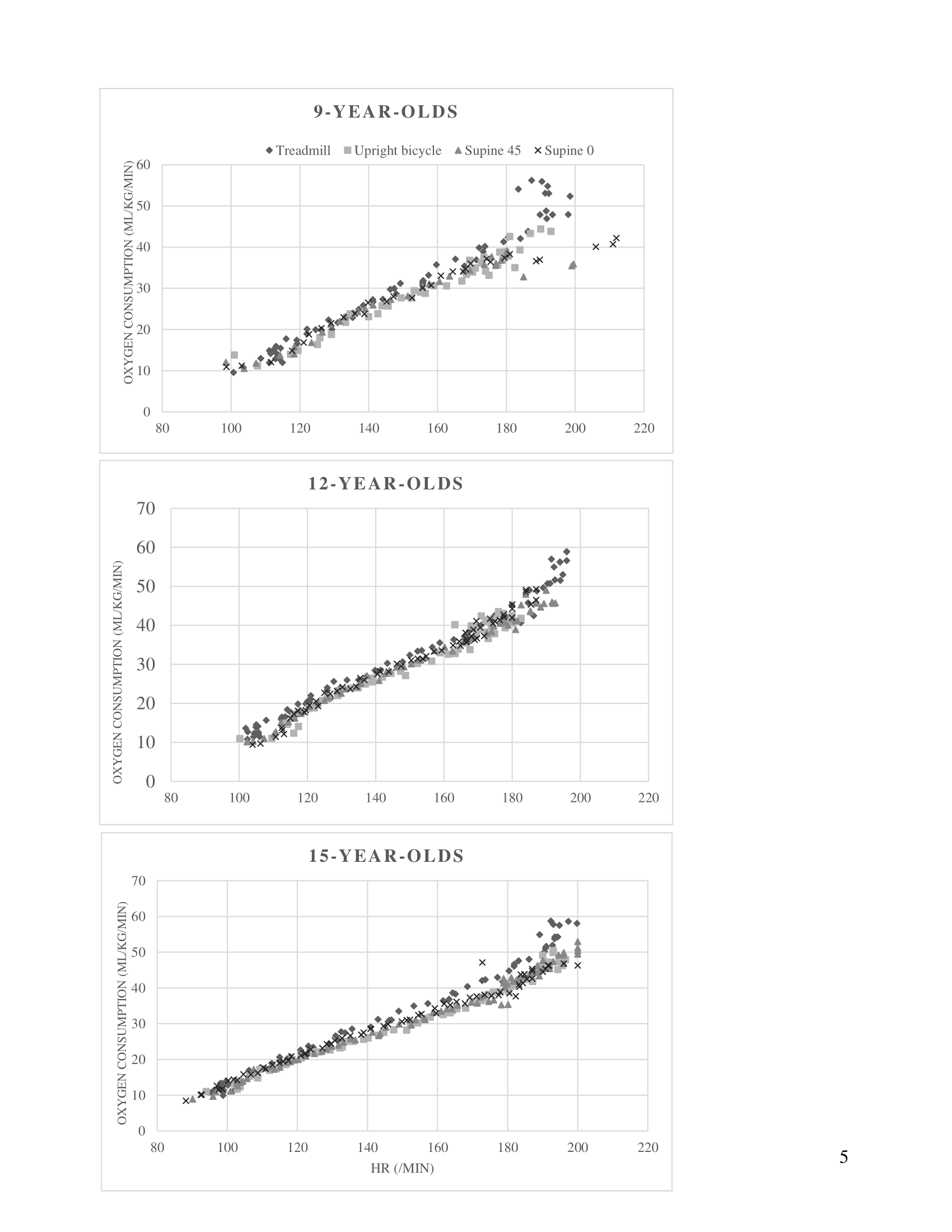

Supplement: Supplementary file 5 [file Image_4.tiff]

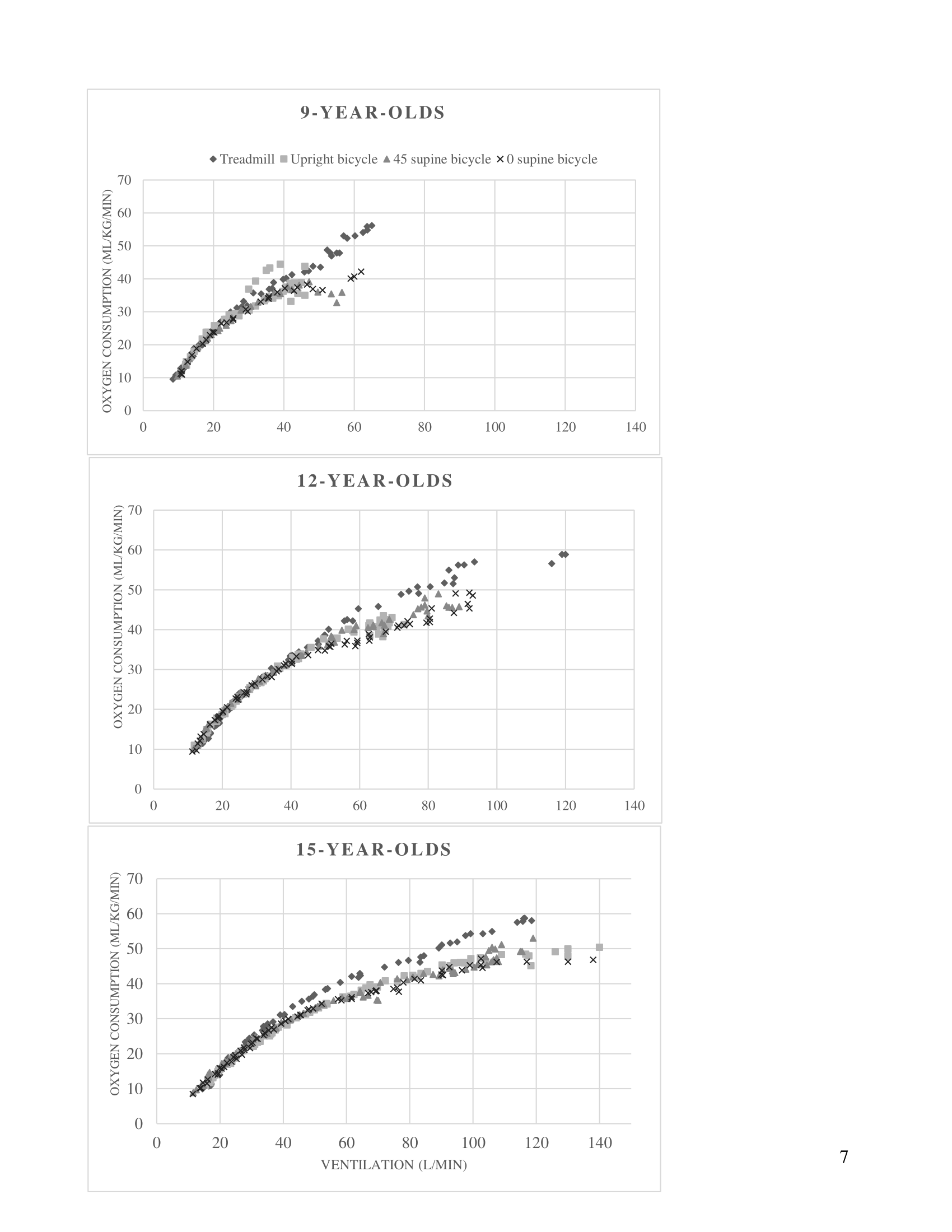

Supplement: Supplementary file 6 [file Image_5.tiff]

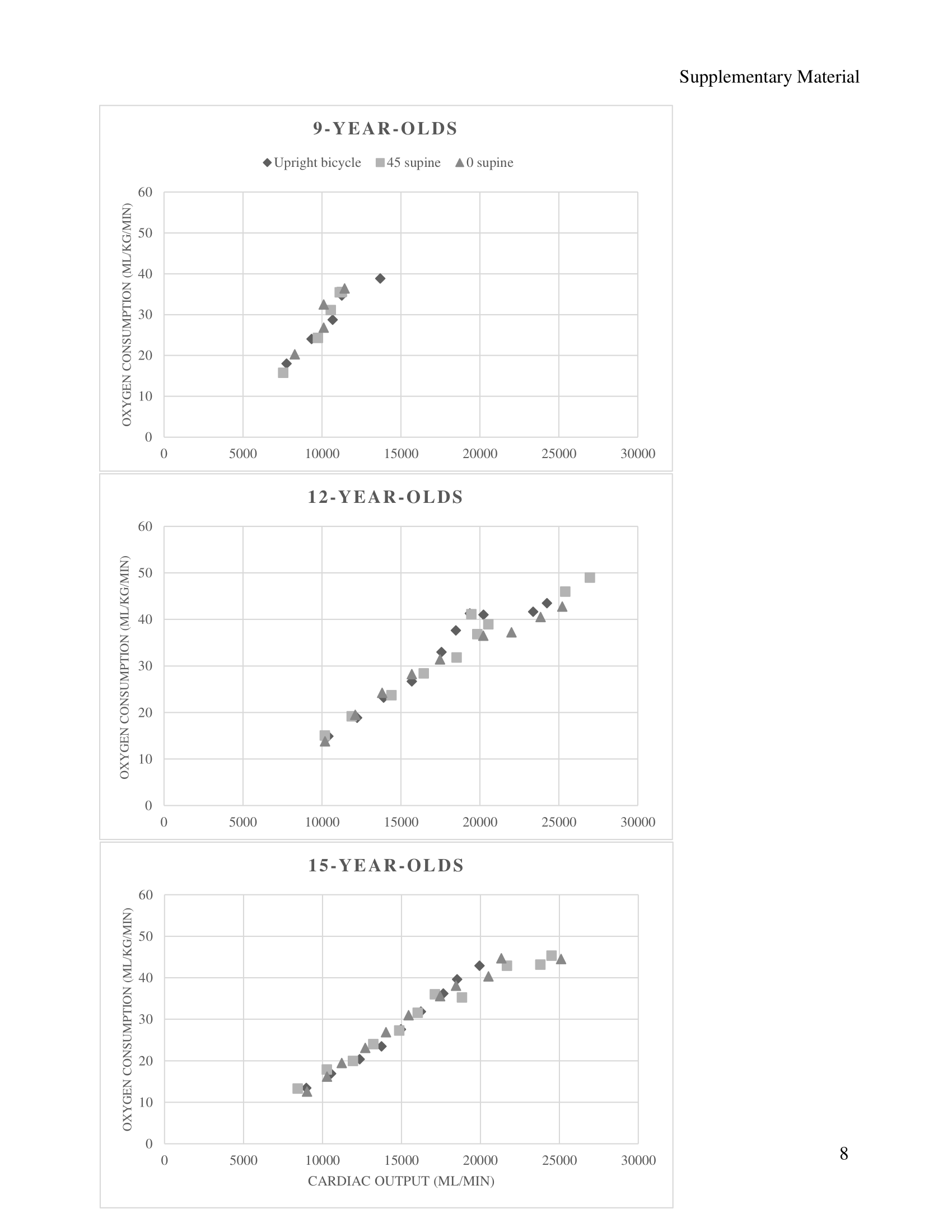

Supplement: Supplementary file 7 [file Image_6.tiff]
